# Biophysical perspectives to understanding cancer-associated fibroblasts

**DOI:** 10.1063/5.0199024

**Published:** 2024-06-06

**Authors:** Somayadineshraj Devarasou, Minwoo Kang, Jennifer H. Shin

**Affiliations:** Department of Mechanical Engineering, Korea Advanced Institute of Science & Technology (KAIST), Daejeon, Korea

## Abstract

The understanding of cancer has evolved significantly, with the tumor microenvironment (TME) now recognized as a critical factor influencing the onset and progression of the disease. This broader perspective challenges the traditional view that cancer is primarily caused by mutations, instead emphasizing the dynamic interaction between different cell types and physicochemical factors within the TME. Among these factors, cancer-associated fibroblasts (CAFs) command attention for their profound influence on tumor behavior and patient prognoses. Despite their recognized importance, the biophysical and mechanical interactions of CAFs within the TME remain elusive. This review examines the distinctive physical characteristics of CAFs, their morphological attributes, and mechanical interactions within the TME. We discuss the impact of mechanotransduction on CAF function and highlight how these cells communicate mechanically with neighboring cancer cells, thereby shaping the path of tumor development and progression. By concentrating on the biomechanical regulation of CAFs, this review aims to deepen our understanding of their role in the TME and to illuminate new biomechanical-based therapeutic strategies.

## I. INTRODUCTION

The tumor microenvironment (TME) is established as a pivotal element in cancer progression within the annals of oncology. Its importance was first noted by Paget in 1889 with the observation of selective metastatic behavior influenced by the surrounding stroma.^1^ This initial observation was elaborated upon by Van den Hooff, who explored stromal engagement in malignancy, highlighting the dynamic interactions within the stromal microenvironment.^2^

Significant advancements in our understanding of TME were made by Sonnenschein and Soto in “The Society of Cells,” where they emphasized the importance of cellular interplay within the TME, laying the groundwork for the Tissue Organization Field Theory (TOFT).^3^ Subsequent research by Park *et al.* delved into the TME's regulatory impact on malignant phenotypes, reinforcing its integral role in the initiation and progression of cancer.^4^ Intercellular and matrix communication has been fundamental in understanding tumor biology. The principle of Stromal Dynamic Reciprocity (SDR) elaborates on the bidirectional and multifaceted interactions between stromal components and cancer cells, which are essential in dictating tumor trajectory.^5–7^

Fibroblasts, integral to maintaining tissue homeostasis and extracellular matrix (ECM) production, undergo a malignant transformation within the TME to become cancer-associated fibroblasts (CAFs). These CAFs are not merely passive elements but active manipulators of the TME, promoting tumor growth, ECM remodeling, invasion, and metastasis.^8–10^ Emerging from various precursors within the TME, CAFs acquire distinctive morphological and mechanical properties pivotal to their tumor-modulating functions.^8–10^

The diversity of CAFs is striking, with distinct subpopulations exhibiting various morphologies, functions, and roles in cancer development. The integration of advanced biochemical and single-cell “omics” tools has broadened our understanding of CAFs.^11^ However, this approach, while insightful, offers a limited perspective of the full spectrum of CAF functions within the TME. Recent bioengineering and biophysical advances reveal that physical forces, in conjunction with chemical signals, are instrumental in dictating cell behavior and intercellular communication.

This review highlights the morphological and mechanical characteristics of CAFs, offering insights into how mechanotransduction and biomechanical sensors intricately contribute to their functional capabilities. Our exploration extends to the dynamic physical interplay between CAFs and stromal cells, elucidating their complex contributions to the orchestration of tumor progression. This discourse not only underscores the significance of the biophysical characteristics of CAFs but also sets the stage for innovative therapeutic interventions in oncology. Additionally, we delved into the potential of biomaterial-based technologies for CAF reprogramming and examined *in vitro* methodologies that replicate the ECM nano-topography, thereby offering refined control over CAF phenotypes and functionalities. By mapping the intricate details of CAF biophysical properties and interactions, this review aims to lay the foundation for future research directions and clinical strategies. The insights presented here have the potential to revolutionize current cancer treatment paradigms, steering toward therapeutic innovations that leverage the mechanical and structural intricacies of the TME.

## II. BIOPHYSICAL CHARACTERISTICS OF CAFS

The biophysical attributes of CAFs are pivotal, given their direct influence on tumor development and progression. Embracing these attributes presents a potential pathway to augment the current method of CAF subtype identification, which relies exclusively on biochemical markers. Each CAF subtype displays unique biophysical traits, such as cellular morphology, motility, contractility, and mechanical properties. These differences often translate into either tumor-promoting or tumor-inhibiting potential.^10^ We provide a comprehensive overview of CAFs’ biophysical traits, categorized into morphology, motility, and mechanical properties.

### A. Morphological variability and motile characteristics of CAF subtypes

CAFs’ morphology and dynamic traits are inherently tied to their gene expression profile. Hence, these characteristics likely serve as comprehensive indicators of CAF function at the cellular level.^12^ Consequently, incorporating both morphological and dynamic characteristics with biomolecular markers could provide a more detailed depiction of the diverse CAF subtypes.

Historical research has identified substantial morphological differences between normal fibroblasts and CAFs. It has been observed that CAFs generally have a larger spreading area,^13,14^ which is often linked to the myofibroblastic characteristics of specific subgroups within the population.^15,16^

Moving away from the traditional view of CAFs as a homogeneous population, Pelon *et al.* have classified heterogeneous CAFs based on their biomolecular markers, adopting a more nuanced perspective to studying CAF biology.^17^ Among the four subtypes of CAFs they identified (CAF-S1 to S4), emphasis was placed on two subsets, which were both myofibroblastic subtypes (CAF-S1 and CAF-S4). Although both subtypes possess myofibroblastic features, distinctive differences in functional characteristics, such as cellular traction forces, were observed. CAF-S1 exhibited a smaller cell spreading area and a higher aspect ratio, while CAF-S4 displayed a broader spreading area and a lower aspect ratio. These observations affirm that CAF morphology is a vital biophysical determinant of CAF behavior.

For instance, a study by Cao *et al.* suggested that the spreading area of CAFs can determine their subtype and function, potentially allowing them to transition between myofibroblastic and inflammatory states through modifications by hydrogel network density.^18^ This study emphasizes the complex nature of CAFs, whose morphological features are just one aspect, necessitating a comprehensive, holistic approach to effectively differentiate CAFs from normal fibroblasts.

The contractile phenotype of CAFs has been extensively explored as a key hallmark of CAF activation.^10,19–25^ Notably, this contractility feature is most prominently observed in a selected CAF subset named myCAFs, a myofibroblast-like phenotype,^18,26–31^ which aligns with the primary functions of CAFs and involves remodeling the ECM through mechanical forces. This contractile behavior of CAFs is governed by a complex interplay of signaling pathways, including Rho-ROCK-myosin, JAK1, and the influence of cytokines like oncostatin M, TGF-β, Calponin 1, as well as the transcription factor YAP.^25^ Additionally, caveolin 1^32^ and α-SMA are implicated in this process,^20,33^ although the exact relationship between α-SMA and contractility remains an ongoing subject of investigation. The degree of α-SMA expression has been extensively investigated and found to correlate consistently with the strength of fibroblast spreading, contractility, and focal adhesion formations in various situations.^34,35^

α-SMA, an actin isoform, serves as the definitive marker for identifying activated fibroblasts, smooth muscle cells, and blood vessels.^35^ It is a standard biomarker for identifying CAFs in the stromal environment and is also a shared marker for myofibroblasts due to their acquired contractile potential.^8,36,37^ The activated state of fibroblasts is distinguished by the presence of stress fibers containing α-SMA, contributing to increased contractility and altering the morphology of activated fibroblasts. CAFs rich in α-SMA expression display a distinctive morphology characterized by oriented stress fibers, augmented lamellipodia, and a concurrent alteration in inherent stiffness. Although α-SMA is associated with cell contraction, it is unclear if α-SMA actively drives contraction or is a passive by-product, as evidenced by studies suggesting TGF-β-induced contraction may occur independently of α-SMA.^38,39^

CAFs have been implicated in modulating the motility and metastatic capability of several cancer types.^40–45^ Moreover, CAFs also acquire different motile behaviors compared to normal fibroblasts. For instance, in gastric CAFs, Ishimoto *et al.* observed an overexpression of RHBDF2, leading to enhanced cell motility.^46^

Similar to morphological diversity, CAFs’ motility also reflects their heterogeneous nature. Costea *et al.* reported that two distinct subtypes of CAFs in oral squamous cell carcinoma (OSCC) manifested varied motile characteristics and different influences on cancer cells.^47^ Notably, CAF-N (similar transcriptomic profile of normal oral fibroblasts) displayed enhanced migratory tendencies compared to both CAF-D (more divergent transcriptomic profile of normal oral fibroblasts) and normal oral fibroblasts. Collectively, these studies illuminate the potential of integrating morphological and motile features of CAFs with biomolecular markers to refine our understanding of CAF subtypes.

### B. Mechanical properties of CAFs

The mechanical properties of CAFs are as heterogeneous as their morphological traits, which arise from their diverse cytoskeletal arrangements. In an effort to understand these differences, the study by Stylianou *et al.* employed atomic force microscopy (AFM) to assess Young's modulus of pancreatic CAFs and normal fibroblasts, finding that myofibroblastic pancreatic CAFs are less stiff than their normal counterparts on a 3 mg/ml collagen substrate (∼14 kPa).^48^ On a softer substrate with a lower collagen concentration substrate (∼4 kPa, 0.5 mg/ml), however, the myofibroblastic pancreatic CAFs and normal fibroblasts exhibited comparable Young's moduli. The differential mechanical sensitivity refers to how each cell type's elastic modulus changes in response to the stiffness of the underlying substrate.^48^

Normal fibroblasts appear to adjust their cellular elasticity more significantly in response to substrate stiffness, whereas myofibroblastic pancreatic CAFs do not exhibit the same degree of sensitivity. This finding aligns with observations that normal fibroblasts develop more aligned stress fibers on rigid substrates, whereas CAFs maintain their stress fiber alignment independent of substrate stiffness. These results may collectively suggest that CAFs, despite varied substrate conditions, retain a consistent biomechanical signature, which could be reflective of their pathological role in the TME.

Further complexities in characterizing CAF biomechanics were noted in the work of Jaeschke *et al.,* demonstrating that prostate CAFs exhibit a higher Young's modulus than normal prostate fibroblasts, with these variations being influenced by factors, such as ECM density and measurement methodologies.^49^ Such differences highlight the need for careful consideration of experimental design when investigating the biomechanical properties of CAFs.

The diversity in biomechanical behaviors observed among CAF subtypes suggests influences from their origin or activation state. To interpret the interconnectedness of morphological and mechanical properties and their collective impact on cancer progression, integrating morphological, motility, mechanical assessments, and transcriptomic analysis is imperative. This comprehensive approach not only aids in understanding the complex interplay between CAF characteristics but also serves as a foundation for identifying and developing therapeutic strategies targeting the biomechanical properties of CAFs to suppress cancer progression.

## III. BIOPHYSICAL INTERACTIONS OF CAFS IN TME

**FIG. 1.**
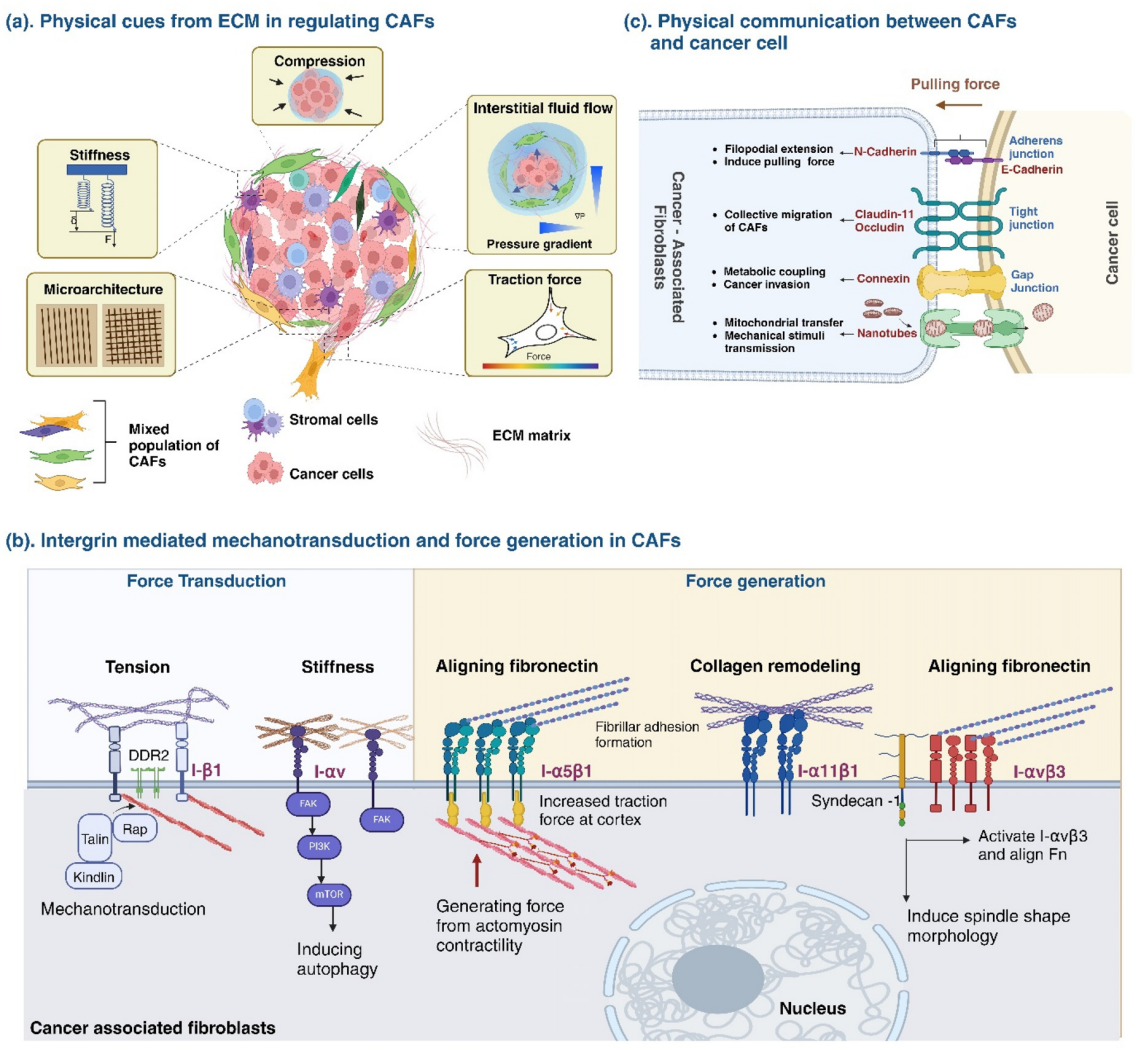
Biophysical perspectives to understanding cancer-associated fibroblasts. (a) Impact of physical cues from extracellular matrix (ECM) in regulating phenotype and function of CAFs. (b) The role of integrin-mediated force generation and mechanotransduction across diverse mechanosignaling pathways that influence gene expression and the heterogeneous functions of CAFs. (c) The dynamic interplay between junctional proteins (JPs), including adherens junctions, gap junctions, tight junctions, and nanotunnels, between CAFs and cancer cells is emerging as a critical determinant in cellular recognition and stromal function regulation. (Schematic was created using BioRender Software.)

Cellular interactions and communication are pivotal in sculpting the TME.^50^ Beyond the inherent biophysical characteristics of CAFs, their interactions with ECM and other neighboring cells are instrumental in TME dynamics. Such interactions shape the TME and significantly influence CAF phenotypes and behaviors. Given that CAFs respond to a multitude of physical cues, alterations in CAF-ECM dynamics, cell-to-cell interactions, and other biophysical elements like ECM stiffness, microarchitecture, compressive stress, and traction forces can modulate CAF activity [Fig. 1(a)]. In this review, we discuss the biophysical interactions of CAFs within the TME, with a specific emphasis on ECM remodeling and cell-cell interactions.

### A. CAF–ECM interactions

CAFs are instrumental in mechanically reconfiguring the ECM, a process that impedes drug penetration through force-dependent mechanisms.^27,51–55^ By contracting their actin cytoskeleton, CAFs generate heightened traction forces, restructuring the matrix and enhancing stiffness.^56^ The reciprocal biophysical interplay between CAFs and the ECM influences CAF behaviors; for example, CAFs respond to integrin-mediated mechanical cues while applying forces that aid ECM transformation.^57,58^

Table I presents a comprehensive overview of CAFs and their critical functions within the TME, highlighting the interaction with the ECM mechanical properties of cancer tissue across various organs, including the lung, liver, breast, bone, and prostate.

**TABLE I.** Key ECM differences and their mechanical properties mediated by CAFs.

Cancer types	Key ECM components	ECM mechanical properties	Characterization	Impact of CAF biology	Clinical implications	References
**1. Pancreatic cancer**	Collagen I, III, IV, V hyaluronic acid (HA), and fibronectin	Increased stiffness and solid stress	Shear wave electrography harmonic motion elastography (HME) harmonic motion imaging (HMI) atomic force microscopy (AFM)	CAFs actively contribute to fibrosis in pancreatic ductal adenocarcinomas (PDACs) by producing enhanced ECM components, particularly collagen and hyaluronic acid (HA), and altering the mechanical properties of the tumor, creating a dense and stiff fibrotic stroma	Dense ECM acts as a barrier, blocking chemo drugs from reaching cancer cells	59–63
		Density	Hematoxylin and eosin (H&E) staining	Pancreatic stellate cells, expressing (α-SMA+), regulate the stromal density by altering collagen fibril organization (loose versus dense packing)	Stromal ECM density modifying agents have direct implications on patients’ outcome	64
		Collagen fiber alignment, fiber dimensions, porosity, and branching global fiber organization	Ultrastructural quantification algorithm and trichrome staining	Inflammatory CAFs create a disorganized tumor microenvironment through pro-inflammatory signals, promoting adverse outcomes by facilitating cancer cell interaction with B cells	Neoadjuvant chemotherapy may promote inflammatory CAFs within the tumor stroma, highlighting the need to understand CAF biology for improved treatment strategies and antifibrotic drugs in PDAC	65
		Highly aligned collagen fiber	Second harmonic generation (SHG) microscopy and advanced digital pathology technologies histology	CAFs, particularly those expressing α-SMA and Syndecan-1, are critical drivers of collagen fiber alignment in the PDAC stroma	Collagen alignment assessment, pre and post-surgery, holds promise for guiding clinical decisions and patient management	66 and 67
		Viscoelasticity	Mesoscale indentation	CAFs promote cancer spread and worsen fibrosis by stimulating collagen and HA production, leading to a fibrotic stroma	Elastography techniques show promise in differentiating PDAC from pancreatitis and normal pancreas based on their mechanical properties	68
		High interstitial fluid pressure (IFP) (HA accumulation)	Pressure catheter piezoelectric probe technique	Not directly assessed	High IFP due to excess HA impedes drug delivery and contributes to therapeutic resistance in PDAC	69–72
**2. Breast cancer**	Collagen I, Fibronectin tenascin-C lysyl oxidase (LOX) matrix metalloproteinases (MMPs)—MMP-1, -7, -9, -11, -12, -14 tissue inhibitors of metalloproteinases (TIMPs)—TIMP-1, TIMP-4	Increased stiffness in the core and heterogeneous stiffness distribution till the periphery	Indentation-type AFM (IT-AFM) Magnetic resonance elastography (MRE)	CAFs promote excessive ECM deposition, potentially via exosomal Cav-1 that increases tenascin-C expression	Excessive ECM deposition, a hallmark of fibrosis, promotes metastasis and poor prognosis, suggesting it as a potential target for therapy	73–78
		Tumor-associated collagen signature -3 (TACS-3)	Multiphoton single harmonic imaging microscopy	CAF induces TACS formation through collagen deposition through syndecan-1	TACS-3 collagen alignment, detectable by SHG or picrosirius red staining, may serve as a prognostic biomarker for breast cancer and a potential target for therapy	79 and 115
		Collagen fiber network density	MRI, Multiphoton second harmonic imaging microscopy, or SHG	α-SMA+ CAFs densify the collagen network, promoting reactive desmoplastic stroma	COX-2 inhibitors may be useful for treating COX-2-dependent cancers by affecting the ECM and CAF activity	80
				Blocking COX-2 in cancer cells reduces CAFs within tumors and metastases		
		Collagen alignment	Masson's trichrome staining	Increased ECM density and alignment promote CAF induction (as measured by α-SMA expression)	Denser and more organized ECM environments activate fibroblasts into CAFs (measured by α -SMA) and suppress T cell activity, potentially fostering an immunosuppressive tumor microenvironment that fuels cancer progression	81
		Highly dense ECM	MRI, multiphoton second harmonic imaging microscopy	CAFs rely on DDR2 signaling to produce and organize collagen fibers, impacting tumor cell invasion	DDR2, a receptor tyrosine kinase expressed in both tumor cells and CAFs, regulates ECM remodeling and promotes breast cancer metastasis	82
**3. Lung cancer**—**Non-small cell lung cancer (NSCLC)**	Collagen I, IV LOXL1 MMP-2, laminin	Increased fibrillar collagen stiffness	AFM	CAFs expressing stromal protein integrin α11 influence tumor growth and spread by affecting collagen stiffness and cross-linking	Blocking integrin α11 in CAFs weakens collagen networks and hinders tumor growth and spread	83
		Desmoplasia	H&E staining	Researchers identified two CAF subpopulations: high desmoplasia (HD-CAFs) and low desmoplasia (LD-CAFs). HD-CAFs remodel the collagen matrix, promoting tumor cell invasion and growth (both in lab studies and animal models)	Targeting functionally distinct CAF subsets might be a promising therapeutic strategy	84
				Notably, a gene called ST8SIA2, highly expressed in HD-CAFs, further enhances cancer cell invasion in 3D models	ST8SIA2 could be a potential target for diagnosis and/or therapy in aggressive NSCLC
		Fibrillar collagen organization and cross-linking	SHG	CAFs express integrin α11, which is critical for promoting collagen organization through LOX cross-linking		83
		MMP-2 degradation of basement membrane likely affects ECM architecture	H&E staining	Enhanced MMP-2 expression in stromal fibroblasts	High MMP-2 expression in tumor cells is associated with an increased risk of recurrence and poor prognosis in NSCLC patients	85
		Basement membrane mechanics: RevMatriRegs—Reversible extracellular matrix regulators that modify the mechanical properties of ECM networks. They adjust the stiffness of the basement membrane (BM) through interactions with laminin. Notably, BM stiffness plays a more critical role than pore size in cancer cell invasion. A softer BM, enriched with RevMatriRegs, such as Net4, impedes cancer cell invasion and metastasis	AFM	CAFs induced BM breaching through mechanical modifications is crucial for metastasis	BM mechanics, particularly stiffness, play a critical role in cancer progression and metastasis	86
				Net4 is primarily produced by CAFs and endothelial cells	High Net4 content in the BM is associated with better patient survival	
					Targeting BM stiffness using RevMatriRegs like Net4 could be a promising therapeutic approach to prevent metastasis	
**4. Colorectal cancer**	Collagen I, III fibronectin, tenascin	Increased stiffness—spatial heterogeneity in the elastic properties of normal and neoplastic ECM increased cross-linking of collagen fibers	AFM, nano-indenter	Increased collagen deposition and remodeling by CAFs, indicated by high αSMA expression near blood vessels, contribute to ECM stiffening	CAF-mediated increased stiffness prognostic marker for CRC-peritoneal metastasis	87–93
				This process, potentially driven by LOX-mediated collagen cross-linking, promotes pre-metastatic niche formation and increased vascularization, creating a favorable environment for cancer cell spread	Targeting tumor stiffness with lysyl oxidase inhibitors might be a therapeutic approach for RAS-mutated CRC	
		Increased collagen content and altered collagen structure (linearized and aligned fibers) in tumor tissues	AFM, Raman microspectroscopy	Stromal cell-mediated enhanced collagen remodeling	High levels of glucasoaminoglycans could be a potential diagnostic endogenous biomarker for early onset pathological ECM remodeling	93
		Stiff, parallelized collagen fibers in colon carcinomas exhibit increased levels of glycosaminoglycans (GAGs)				
**5. Ovarian cancer**	Collagen I	Collagen abundance, fiber orientation, and architecture	Masson's trichrome stain	DDR2 signaling in CAFs regulates collagen production through arginase-1	Targeting DDR2 or arginase-1 in CAFs may be a potential therapeutic strategy for ovarian cancer by affecting collagen production	94
**6. Prostate cancer**	Collagen I tenascin,	Increased deposition of collagen I	H&E staining	Myofibroblasts mediated collagen remodeling	Collagen I expression is likely to be a key feature of reactive stroma in tumorigenesis	95

Alterations in ECM stiffness initiate mechanosignaling pathways that activate CAFs, fostering a dynamic and active stroma contributing to tumor progression.^24,57,96,97^ Moreover, CAFs exert mechanical forces on the ECM, modifying the orientation and organization of ECM fibers.^27,52,98^ Integrins are vital in communicating these stiffness-related signals to the cell nucleus, where CAFs, through integrin-mediated contractility, exert forces that influence matrix alignment and clustering.^99–102^ CAFs establish a self-reinforcing loop by maintaining activation through a persistent contractile phenotype, transforming the ECM into a highly reactive desmoplastic stroma.^96,100,103^ Continuous contraction of CAFs and adopting a contractile form generate mechanical forces via integrins. These forces increase the stiffness of the ECM and activate latent TGF-β1, a crucial player in fibrotic remodeling and stromal activation. This activation triggers the deposition of collagen and fibronectin, causing a rise in ECM rigidity and shifts in its composition. The extensive remodeling and breakdown of the ECM affect interactions between cells and the ECM, altering the abundance and composition of fibronectin and collagen. Consequently, this process reduces the spacing between ECM networks and ligands, impacting cell-ECM interactions. Integrins play a bidirectional role in coordinating this mechanical modification of the ECM.

#### 1. Integrin-mediated mechanosensing and force generation in CAFs

Integrins are pivotal in CAF activation, mechanotransduction, and force generation, thus playing a crucial role in modulating the TME and influencing cancer progression. Various integrins (α3β1, α5β1, α11β1, αvβ1, αvβ3, αvβ5, αvβ8) interact with ECM components like fibronectin, periostin, TGF-β1, laminin-322, and collagen, impacting the TME's structure and dynamics,^27,104–108^ thereby affecting the TME's structure and function. Integrins function as mechanosensors that detect changes in ECM stiffness and exert mechanical forces, which are instrumental in tumor invasiveness and the remodeling of the matrix.

Mechanotransduction involves integrins sensing ECM stiffness through mechanisms, such as the recruitment of Talin1 and Kindlin2 by Discoidin Domain Receptor 2 (DDR2), leading to collagen-binding integrin activation.^57^ Additionally, αv integrins facilitate stiffness perception and cellular response through focal adhesion kinase, triggering processes like AMPKα activation and autophagy, supporting adjacent cancer cell growth.^109^ A comprehensive overview of the myriad integrins and their roles in mechanotransduction within CAFs is listed in Table II.

**TABLE II.** List of integrins that regulate mechano-transduction and force generation in CAFs.

Integrins	Experimental model	Activation pathway	CAF function	References
β1	Breast tumor organoids	DDR2 regulate the β1 Integrin through activation of Rap1	Mechano-transduction	57
αv	PDAC	αV-FAKs sense and transduce the mechanical signal to AMPK and stabilize mTOR	Stiffness-mediated stromal autophagy	109
α5β1	SCC	Integrin α5 accumulates at the plasma membrane through the aid of Rab 21 protein	Stimulates actomyosin contractility to remodel the matrix	14
	Prostate	Enhanced myosin-II-driven contractility and increased traction forces in CAFs were transduced to the ECM by Int α5β1	Fibronectin fibrillogenesis, force-mediated ECM remodeling, and mediate directional migration of cancer cells	23 and 110
α11β1	Non-small cell lung carcinoma (NSCLC)	Collagen binding ECM receptor	Differentiation of fibroblasts into CAFs	83
			Mediate collagen cross-linking and increasing stiffness	29, 83, 105, and 111–113
	Lung cancer spheroid model	Mediate autocrine expression of CXCL5 in lung cancer cells	Regulate fluid pressure between tumor structures by remodeling collagen	
αvβ3	Colon tumors	⋯	Regulate contractility and trigger fibrillary adhesions and force-mediated Fn assembly	114
	Breast cancer cell line	Sdc1 activates integrin αvβ3.	Sdc1 induces spindle shape morphology and produces aligned Fn fibers through activation of integrin αvβ3	115 and 116

In force generation and matrix remodeling, α5β1 integrin enhances cancer cell invasion via ECM remodeling, while α11β1 integrin, upregulated in desmoplastic tumors, promotes cancer cell invasiveness through force-dependent contractility and collagen remodeling.^23,28,83,110,111^ αv integrins, especially αvβ3, influence fibrosis, directional migration, and cancer cell invasion by mediating contractility, fibrillary adhesion formation, and fibronectin fibrillogenesis.^23,114^ Syndecan-1 (Sdc1) cooperates with αv integrin in these processes, influencing ECM fiber alignment and cell morphology.^114–119^ A list of integrins involved in CAF force generation can be found in Table I. Integrin-mediated force generation is key in matrix stiffening, a hallmark feature of aggressive tumors, where integrins convey cellular contraction forces to the matrix, aiding in its organization and enhancing cancer cell migration.^52,112,120^ Additionally, CAF-secreted enzymes, like lysyl oxidases (LOXs), contribute to matrix cross-linking and stiffening, further contributing to desmoplasia by cross-linking collagen fibers.^121,122^

Future investigations should focus on the diversity of integrin-mediated force generation in mechanosensing pathways and its effect on gene expression and CAF subtype functions. Employing biophysical techniques and single-cell analysis will elucidate the heterogeneity and its implications on stromal biomechanics.

#### 2. Fluid flow and CAF phenotype

The role of fluid flow within the TME is an underappreciated yet critical determinant of the CAF phenotype, orchestrating cell behavior through the transit of nutrients, metabolic waste, and signaling molecules.^123–125^ Interstitial fluid flow (IFF), though characterized by low velocity, exerts a substantial influence on CAF activity. It triggers myofibroblast differentiation, ECM remodeling, and stiffening, attributed mainly to TGF-β signaling.^126,127^ Fluid shear stress, comparable to the mechanical forces exerted by solid stress, fosters the development of a denser and more intricate matrix network. While hindering fluid transport, this network paradoxically enhances cell motility, likely due to the creation of differential shear gradients across cells.^127^

Recent studies employing 3D cultures and microfluidic models have shed light on how IFF influences CAF behavior,^128–132^ revealing its role in promoting Endothelial to Mesenchymal Transition (EndMT) during CAF differentiation. Constant unidirectional IFF not only drives fibroblast migration toward cancer cells but also correlates with increased expression of CAF markers, such as MMP-9, MMP-14, FAP, vimentin, and α-SMA.^131^ Moreover, IFF-induced shear stress activates normal fibroblasts into CAFs through the IGF-PI3K signaling axis, emphasizing the fluid flow's influence on CAF function and differentiation.^133^

CAFs are not mere bystanders to IFF within the TME but also active regulators. The integrin α11β1, expressed by CAFs for instance, is noted for its regulatory capacity on interstitial fluid pressure, influencing the intensity of IFF across various cancers, possibly by influencing the collagen network.^134^ Such dynamic flow can distort chemokine distribution, enabling the guided migration of cancer cells and CAFs, thereby supporting invasive behavior and metastatic potential. Additionally, the presence of CAFs alongside circulating tumor cells (CTCs) in the bloodstream invites speculation on their possible role in modulating IFF, potentially impacting tumor cell behavior in metastatic niches.^135,136^

In essence, CAFs are both shaped by and shaping the fluidic landscape of the TME. Their reciprocal relationship with IFF commands further exploration to fully grasp the influence of fluid mechanics on the nuanced roles of CAF subtypes in the evolution and dissemination of cancer.

### B. Mechanical communication between CAF and stromal cells

Historically, the emphasis on cell-to-cell communication has centered on chemical signaling. However, newer research underscores the significance of physical interactions in these communications.^21,118,137,138^ The dynamic interplay between junctional proteins (JPs), including adherens junctions, gap junctions, tight junctions, and nanotunnels, between CAFs and cancer cells is emerging as a critical determinant in cellular recognition and stromal function regulation [Fig. 1(c)], Table III.

**TABLE III.** List of junctional proteins in CAF-mediated mechanical communication in stroma.

Junctional proteins	Experimental model	Context of activation	CAF function	References
N-Cadherin	Colon cancer	rTGF-β1 upregulates N-cadherin expression through JNK activation	Filopodia formation, polarized morphology	139
	Human lung adenocarcinoma		Induce pulling force to the dissemination of cancer cells	140
OB-Cadherin	Wound healing	Binding to α-SMA	Mechanical stability of myofibroblasts population	141
Occludin and claudin	Colon cancer	TGF-b1 dependent pathway	Collective migratory pattern of CAFs	142
Connexins	NSCLC	Cx43 form unidirectional connexins	Metabolic coupling and invasion of cancer cells	143 and 144
Tunneling nanotubes	Breast cancer cell lines	Increasing mitochondrial oxidative phosphorylation	Reprogramming and transmission of mechanical stimuli	145
			Increase 3D migration of cancer cells	

#### 1. Adherent junctions of CAFs

Adherens junctions (AJs) primarily facilitate the transmission of intercellular forces via direct cell-to-cell contact, forming mechanical links between the cytoskeletons of adjacent cells.^146^ Within CAFs, cadherins like N-cadherin and OB-cadherin are present and linked with fibroblast differentiation and high contractile activity.^141^

Different cell types express cadherins, such as E-, N-, and P-cadherin, each playing a distinct role in transmitting intercellular forces among stromal cells. The upregulation of N-cadherin in myofibroblasts, including CAFs, promotes directional migration and filopodia formation while also facilitating mechanical linkage with cancer cells or other stromal cells, thereby enhancing their ability to migrate and invade.^139^ Filopodia and lamellipodial-like structures enable direct mechanical communication between CAFs and stromal cells, as observed in two-dimensional cultures.^147^ Notably, CAFs and cancer cells can form heterotypic adhesions between E-cadherin and N-cadherin, promoting the transfer of intercellular forces and furthering cancer cell invasion.^140^

Cancer cells can suppress p53 in surrounding fibroblasts, leading to the development of CAFs and promoting cancer invasion through direct cell-to-cell contact. This suppression of p53 may involve TSPAN12-mediated β-catenin signaling within the fibroblasts. Additionally, TSPAN12 upregulates the secretion of the CXC chemokine ligand 6 (CXCL6), which effectively promote invasion. These findings suggest that TSPAN12 and CXCL6 are potential therapeutic targets to disrupt CAF-mediated lung cancer progression.^148^ Direct physical interactions between CAFs and cancer cells, or other stromal cells, significantly regulate CAF and cancer cell phenotypes and functions.^43^

Upon activation, fibroblasts exhibit mechanical coupling through AJs, enabling the transmission of local contractile events to adjacent activated myofibroblasts. This contraction creates a mechanical feedback loop that coordinates and recruits connected cells, promoting tissue remodeling and providing information on the contractile status of neighboring cells.^149,150^ During the transformation of fibroblasts into myofibroblasts, the composition of changes in AJs, with OB-cadherin replacing N-cadherin. This switch allows OB-cadherin to bind with α-SMA, reinforcing the mechanical stability of the myofibroblast population.^141^ This switching of cadherins may also play a role in the interactions between CAFs and other stromal or cancer cells. The expression of nectin-1, a Ca2^+^-independent cellular adhesion molecule, in CAFs has been correlated with heightened invasion, metastasis, and reduced overall survival in PDAC patients.^151^ This highlights the potential involvement of CAFs in modulating cell–cell adhesion strength through intercellular contact composition and differential cadherin expression ratios.

#### 2. Tight junctions (TJs) in CAFs

Exploring the presence of TJs within stromal populations in desmoplastic lesions and their potential role in supporting barrier functions in cancer therapies offers intriguing research opportunities. In a study by Karagiannis *et al.,* occludin, a TJ protein, was discovered in CAF cohorts within desmoplastic lesions of colorectal cancer patients, suggesting that fibroblasts may undergo a context-dependent switch to express claudin-11, potentially contributing to a more collective migratory behavior.^142^ This intriguing discovery raises the possibility that CAFs harness TJ's machinery to adopt a collective configuration and migrate together as cohorts, potentially influencing tumor behavior.^22^ Moreover, the crucial role of TJ strands in fibroblasts, acting as a fence-like structure, cannot be overlooked as they establish a physical barrier between the apical and basolateral membranes, contributing to the maintenance of barrier integrity.^152^ As cancer therapies often aim to breach these barriers, understanding TJs within stromal subsets could pave the way for innovative strategies that target tumor expansion by dismantling these protective barriers.

#### 3. Gap junctions and tunneling nanotubes in CAFs

Gap junctions (GJs), facilitated by connexins, pose challenges in overcoming chemotherapy resistance within the TME, forming physical barriers between CAFs and cancer cells that compromise treatment effectiveness.^143,144^ Decoding their role in mechanical interactions and metabolic shifts between CAFs and cancer cells is crucial for addressing chemo-resistance.^146^

In the realm of intercellular communication, tunneling nanotubes (TNTs) have emerged as pivotal conduits, connecting distant cells and facilitating the exchange of genetic material.^145,153^ While CAFs predominantly provide resources to fuel cancer cells, cancer cells exploit TNTs to influence CAF behavior.^145^ Deciphering the mechanisms behind TNT formation elucidates their profound involvement in promoting or inhibiting tumor growth. However, their fragile nature renders TNTs vulnerable to mechanical disruptions, potentially compromising their intercellular connections. Understanding the mechanics behind TNTs reveals their elastic nature and the importance of stability and actin elongation.^154–156^ Moreover, these TNTs may relay mechanical signals, affecting cell migration and function. An in-depth understanding of the mechanotransduction processes linked to TNTs in CAF-cancer cell interactions offers insight into their dual role in promoting and inhibiting tumor progression.

Effective communication is the cornerstone of the dynamic interplay between CAFs and their stromal counterparts. AJs, TJs, GJs, and TNTs emerge as key players in orchestrating the exchange of essential signals. The altered expression of JPs by CAFs adds complexity to their dual roles as promoters and inhibitors of tumor growth. The interplay of JPs with actin stress fibers intricately shapes CAF morphology during high-stakes, force-driven activities. A potent biomarker can be identified to decode distinct CAF subtypes by unraveling the enigma of JP expression patterns during CAF development.

### C. Physical interactions of CAFs with inflammatory cells

Although the physical interaction between CAFs and inflammatory cells is often overshadowed by biochemical pathways, it has recently been revealed to be a vital regulator of tumor dynamics. Tsoumakidou's comprehensive review describes major subtypes of CAFs, such as myCAFs, iCAFs, and apCAFs.^157^ The review highlights their impact on the infiltration of immune cells, emphasizing the need for additional research to explore their specific interactions.

MyCAFs play a role in the formation of a dense ECM network, which acts as a physical barrier that restricts the entry of immune cells, specifically the effector T cells that are crucial for combating tumor growth. The myCAFs-ECM-T cell triad deserves closer examination from a biophysical perspective, as it has a substantial impact on regulating T cell activation, clonal expansion, trafficking, and exhaustion.^158–161^

Specifically, ECM proteins secreted by CAFs, such as βig-h3, create obstacles for T-cell infiltration by interacting with integrin β3 on CD8+ T cells. This interaction hinders the immune response against tumors.^162^ However, a promising approach to overcome this CAF-mediated barrier involves the use of T cells armed with membrane-anchored and tumor-targeted IL-12, particularly attIL12 cells. These engineered T cells likely achieve this by stimulating the production of IFNγ upon interaction with tumor cells. IFNγ, in turn, suppresses growth factors secreted by CAFs, leading to CAF apoptosis and a remodeled tumor microenvironment. This remodeled environment allows for better infiltration of effector T cells, overcoming the barrier previously established by the ECM and CAFs.^163^

The phenotypic adaptability of myCAFs has been highlighted in recent investigations. In response to the dense ECM, myCAFs have been observed to undergo morphological transformations, adopting a more rounded shape and shifting toward a high IL-6 secreting profile akin to iCAFs within three-dimensional physio-mimetic culture systems.^18^ This plasticity introduces additional complexity to the myCAF/iCAF-ECM-T cell interface, suggesting that myCAFs, after contributing to ECM densification, may transform into iCAFs, with implications for T-cell interaction and suppressed anti-tumor activity. iCAFs typically express a cytokine-rich, ECM-deficient profile, suggesting a possible plateau in ECM production following their transition from myCAFs, which could further complicate immune cell infiltration. The differing adhesive molecule expressions between iCAFs and myCAFs could affect their physical engagement with T cells, altering T cell activation, mobility, and efficacy within the TME. Tsoumakidou also posits that myCAF and iCAF phenotypes might not be distinct entities but rather extremes on a continuum of fibroblastic differentiation.

**FIG. 2.**
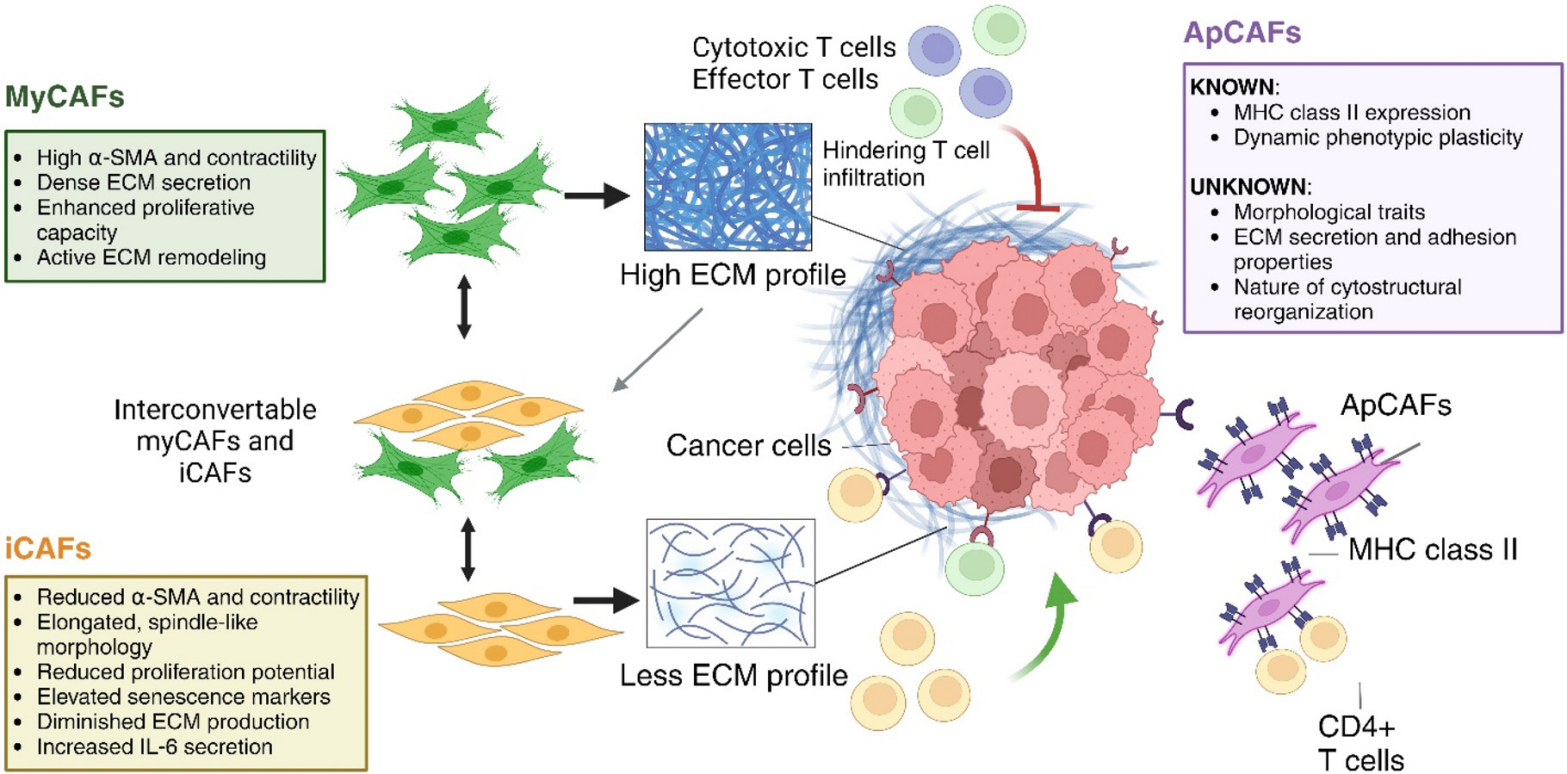
Physical interaction between MyCAFs, iCAFs, and ApCAFs with ECM-T cells axis: MyCAFs secrete a dense ECM network that hinders T-cell infiltration. iCAFs exhibit a lower ECM density and potentially altered adhesion interactions with T cells. apCAFs, expressing MHC-II molecules, can present antigens to CD4+ T cells. Arrows indicate the reciprocal communication between these components, influencing T cell movement and function. (Schematic was created using BioRender Software.)

Furthermore, Tuveson and colleagues have unveiled antigen-presenting CAFs (apCAFs) bearing MHC class II in pancreatic cancer.^164^ These apCAFs, when studied *ex vivo*, may lose MHC II expression and take on myCAF-like characteristics, underscoring the necessity of investigating apCAFs within their native tumor context for their role in sustaining effector CD4+ T cell responses.^157,164,165^ Future studies should dissect the functional distinctions between classical antigen-presenting cells (APCs) and apCAFs, with a specific focus on the adhesion mechanisms governing their interaction with T cells. Physical interaction between CAFs and T cells is illustrated in Fig. 2.

## IV. THERAPEUTIC STRATEGIES TARGETING BIOPHYSICAL PROPERTIES OF CAFS

### A. Current approaches to targeting CAFs in cancer therapy

Targeting the intricate interactions between CAFs and tumor cells revolutionizes cancer treatment, offering new prospects for enhanced patient recovery.^10,31,166–169^ Therapeutic targeting focuses on mechanisms triggering CAF activation and associated signaling pathways.^121^ Numerous signaling cascades significantly impact the biological activities of CAFs and the interplay between CAFs and cancer cells. For example, both the canonical and noncanonical TGF-β signaling pathways in CAFs hold potential as targets, especially concerning their crosstalk with tumor cells. Similarly, the PI3K/AKT/mTOR, Wnt, MAPK, and JAK/STAT pathways are intensively targeted by simultaneously suppressing the crosstalk of CAF with cancer cells. Numerous reviews have already detailed the therapeutic targeting of CAF-mediated signaling pathways with a current list of clinical trials.^10,26,121^ Additionally, innovative strategies, such as CAF depletion, inhibition, infiltration disruption, reprogramming, and the development of CAF-directed therapeutics, vaccines, and immune-based technologies, are under investigation to counteract CAF-mediated tumor progression. The TME and downstream effectors of CAFs are under intensive investigation for potential new treatments.^166^

Despite these advances, the versatile nature of CAFs, which can shift between different subtypes, presents a challenge in enhancing tumor control. Deep insights into the balance between tumor-promoting and restricting CAFs and their dynamic interactions in the TME are essential as they uncover potential targets. CAFs experience significant biophysical shifts, adopting a tumor-promoting phenotype characterized by morphological changes, enhanced migration, and increased force generation. Consequently, strategies focusing on the biophysical regulation of CAFs are promising in cancer therapy. This section provides a detailed look into methods for altering the biophysical properties of CAFs, harnessing their pro-tumorigenic potential, and unlocking their anti-tumor capabilities.

### B. Potential of targeting biophysical cues to regulate CAFs

Reducing contractility and mechanosensing in CAFs contribute to the disruption of the mechanical feedback loop, resulting in reduced desmoplastic reactions and ECM remodeling, thereby suppressing cancer invasion.^170^ In a study conducted by Chronopoulos *et al.*, all-trans retinoic acid (ATRA), an active metabolite of vitamin A, restores mechanical quiescence in pancreatic stellate cells (PSCs) by downregulating actomyosin contractility mediated by the retinoic acid receptor beta (RAR-β).^171^ ATRA treatment in three-dimensional (3D) organotypic models diminishes the ability of CAFs to generate high traction forces, adapt to mechanical cues from the surrounding environment, and inhibit force-mediated ECM remodeling. ATRA has now emerged as a promising stromal targeting agent in combination with chemotherapy for PDAC.^172^

Inhibiting focal adhesion kinase (FAK) activity and targeting proteins involved in cellular contractility, such as myosin II or Rho-associated protein kinase (ROCK), helps deter force generation in CAFs.^170^ When combined with chemotherapy, FAK inhibitors induce a significant decrease in metastasis in PDAC by reducing CAF force generation and ECM remodeling.^173^ Similarly, inhibiting ROCK, a downstream effector of the Rho GTPase that regulates cell contractility, disrupts the contractile properties of CAFs and impacts their functional interactions with cancer cells. ROCK inhibitors hold great promise as therapeutic targets due to their involvement in stromal remodeling, stiffness sensing, contraction, and the formation of stress fibers in CAFs.^169^ Phase I trials investigating the use of ROCK and FAK inhibitors have highlighted the importance of targeting contractility and force-mediated ECM remodeling.

Targeting key signaling pathways involved in mechanotransduction, such as the YAP/TAZ pathway or the Hippo pathway, holds the potential to disrupt the activation of CAFs and mitigate their pro-tumorigenic effects.^174,175^ Activated YAP in CAFs, known for promoting malignancy and angiogenesis, contributes to increased fibrosis surrounding tumor cells. The mechanoactivation of YAP/TAZ in CAFs facilitates feed-forward interactions and impedes the dynamic interplay between CAFs and other cells within the tumor ecosystem.^25,176^ Systematically inhibiting YAP/TAZ in CAFs emerges as a potentially effective treatment strategy to prevent metastasis and overcome chemoresistance.^175^

Inhibiting enzymes involved in ECM remodeling, such as matrix metalloproteinase (MMPs) and LOXs, have shown potential.^26,171^ While preclinical models have demonstrated promising results with MMP inhibitors in preventing CAF-mediated ECM stiffening and desmoplasia,^177,178^ LOX2 inhibitors have also yielded encouraging results in PDAC trials.^179^ However, LOX2 inhibitors have shown limited improvements in patient survival. This discrepancy could be attributed to the multifaceted role of CAFs and the necessity for precisely identifying CAF phenotypic variations associated with LOX2 secretion.

Recognizing that these inhibitors predominantly target CAFs characterized by high contraction and force generation is crucial, neglecting the importance of addressing CAF subtypes lacking these physical traits. Therefore, it is imperative to understand CAFs comprehensively based on their phenotypical signatures, including their physical characteristics. Simplistic pharmacological normalization might not suffice to address challenges within the TME.

In our comprehensive review of the biophysical regulation of CAFs, we emphasize the immense potential of modulating biophysical cues to alter CAF behavior. Furthermore, we explore *ex vivo* biomaterial-based platforms capable of precisely regulating the biophysical traits of CAFs.

### C. Biomaterials in understanding and modulating CAF function

**FIG. 3.**
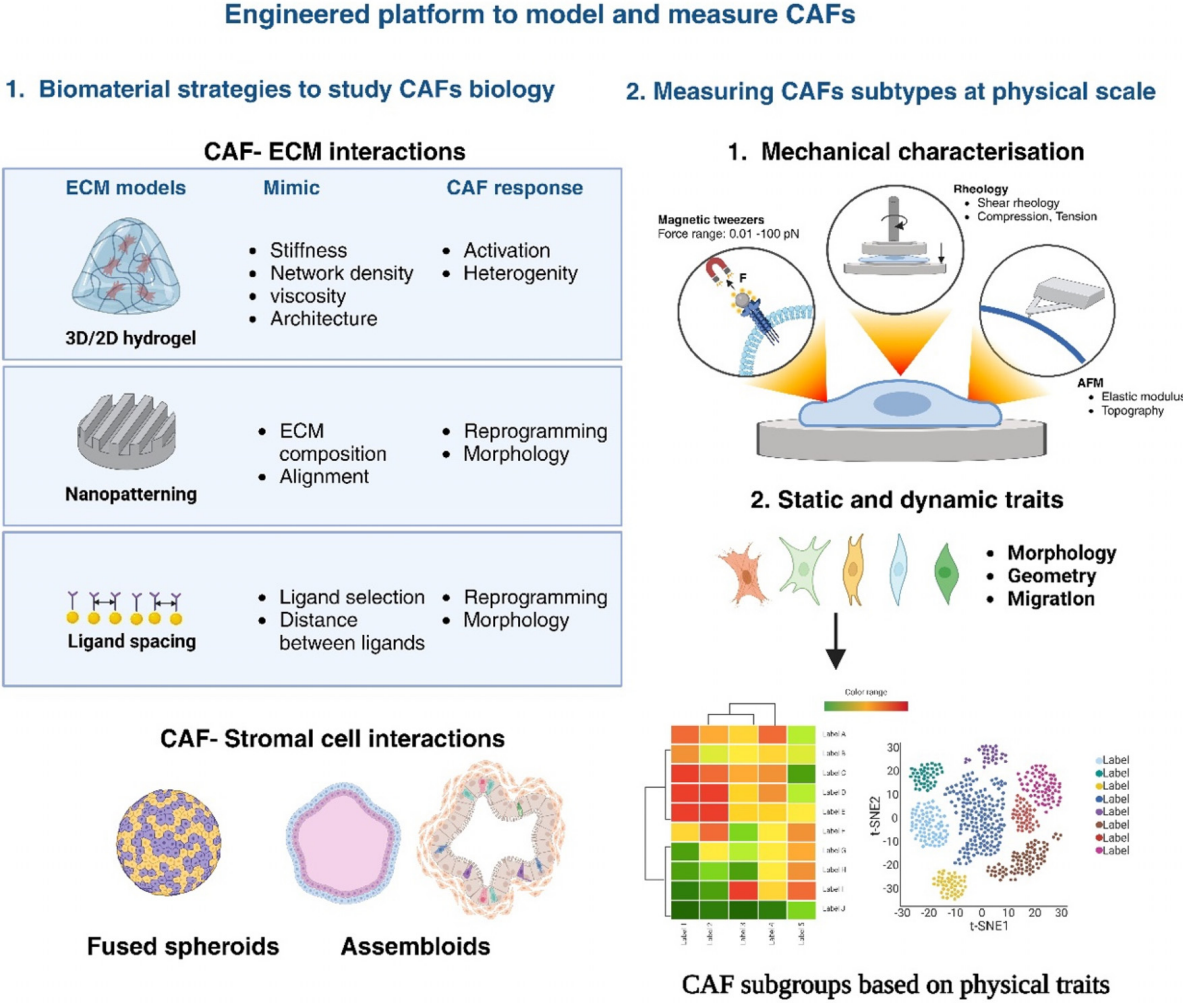
Engineered platforms for modeling and measuring CAFs. A multifaceted approach that includes biomaterial strategies, advanced cellular models, and mechanical characterization tools to model and measure the distinct behavior of CAFs to uncover novel therapies. (Schematic was created using BioRender Software.)

Extensive studies have focused on harnessing the potential of biomaterials to regulate cell fate, both *in vitro* and *in vivo*.^180^ These materials offer a unique opportunity to manipulate both the biochemical and biophysical properties, influencing crucial cellular processes, such as morphology, migration, and differentiation potential, as illustrated in Fig. 3. We highlight specific mechanical properties, including biomaterial stiffness, ECM network density, ligand density, and ligand patterning to steer the phenotypic behavior of CAFs *ex vivo*.

Emerging studies utilizing the collagen and alginate-interpenetrating network (CoAl-IPN) hydrogel system have provided significant insight into the mechano-mediated regulation of CAF behavior and their interaction with tumor cells. For instance, in breast CAFs, a compliant hydrogel network facilitates spreading and augments pro-tumorigenic activities by activating YAP/TAZ mechanosignaling pathways and upregulating CAF-specific genes. In contrast, a stiffer and more cross-linked hydrogel network can constrain the spreading and phenotype of breast CAFs (b-CAFs), subsequently diminishing their pro-tumorigenic paracrine functions.^18,181^

A related investigation on colorectal CAFs demonstrated that variations in the network density of the CoAl-IPN can lead to a reversible phenotypic shift between inflammatory and myofibroblastic states. This shift is governed by the polymer network's confinement effect and the ROS-HIF1-α mechanotransduction-signaling axis. The myofibroblastic state prompts epithelial-mesenchymal transition (EMT) in colorectal adenocarcinoma cells, aiding their dissemination and potentially accelerating tumor progression, whereas the inflammatory state is associated with anti-tumorigenic effects and improved patient survival outcomes in colorectal cancer.^18^

The inflammatory state, indicated by rounded cell morphology, reduced alpha-SMA levels, and elevated IL-6 levels, may be prompted by physical constraints within the tumor stroma. This environmental interaction can induce a transition from pro-tumorigenic, spindle-shaped myCAFs to rounded, potentially tumor-suppressive iCAFs, underlining the significance of physical cues in influencing CAF phenotypes toward tumor suppression.

However, the therapeutic manipulation of CAF morphology and biomechanical properties requires a complex understanding of the distinct physical attributes associated with their tumor-promoting and suppressing functions. It is crucial to identify these characteristics precisely to prevent inadvertently diminishing the beneficial actions of certain CAF subtypes. Ongoing and future research should focus on delineating the specific physical features of CAFs along their functional continuum and understanding how these aspects relate to tumor dynamics. Achieving this will be crucial for developing targeted therapeutic strategies that judiciously modulate CAF functions, supporting their tumor-suppressive capacity while preserving their advantageous roles within the tumor microenvironment.

In a related study conducted by our group, we found that fibrous architecture with high network density in gelatin fibers confines normal fibroblasts, preventing their activation into CAFs by limiting cell spreading and altering morphology.^182^ These findings collectively emphasize the critical role of 3D network density and spatial cellular confinement as key biophysical determinants that profoundly influence CAF phenotypic plasticity and activation. Modulation of cell-ECM binding interactions regulates a spectrum of cellular functions, including motility, differentiation, growth, and morphological responses. Traditional analyses of cell-matrix dynamics often overlook the inherent viscoelastic properties of tissues, which are crucial for accurately recapitulating tissue mechanics across various pathological states, as elucidated by Chaudhuri *et al.*^183^ Our focus encompasses the role of such properties in CAF behavior, particularly within the ECM's interstitial niches.

Recent work by Lin *et al.* utilizing a biomimetic hydrogel system designed to emulate the stiffness and viscoelastic properties of pancreatic cancer tissue revealed that increased matrix stiffness, irrespective of viscoelastic changes, correlates with a reduction in activated CAF markers.^184^ The study further noted that dynamic stiffening of the matrix curtailed CAF spreading and surface area, implying a limitation in their migratory and contractile abilities. Although there were observable shifts in cell cycle-associated gene expression, CAF proliferation remained notably unchanged.^185^ This suggests that replicating dynamic stiffening observed in tumor microenvironments could modulate CAF activity, downregulating activation markers and confining cellular expansion.

Additionally, an earlier study by the same research team emphasized the influence of ECM composition, observing that increased stiffness in conjunction with hyaluronic acid buildup fostered a more elongated CAF morphology, possibly due to enzymatic expression changes.^186^ This finding underscores the intricate relationship between matrix viscoelastic properties and CAF function. By mimicking the native ECM and manipulating its mechanical attributes, biomaterials present promising avenues for mitigating the pro-tumorigenic functions of CAFs, offering potential for innovative cancer therapies.

CAFs form fibrillary adhesions while remodeling fibronectin (FN). Mechanical cues highly regulate the assembly of these adhesions. The contractile forces produced by CAFs can unfold FN, creating tension and revealing cryptic binding sites. FN remodeling, influenced by tension, changes ligand binding site arrangements and overall ECM organization.^187^ While the nanoarchitecture of the ECM is well established on biomaterial platforms, understanding the nanoscale distributions of ligand binding sites that are crucial for stromal cell behavior, particularly CAFs, remains limited. Researchers have crafted tailored ligand presentation platforms at varying scales and spacing to unravel the impact of the spatiotemporal organization of ligands.^188,189^ This approach, extensively studied in stem cells and cancer cells for differentiation and chemoresistance functions, offers a unique opportunity for understanding CAF behavior. As force-generating cells, modulating ligand density at different scales can reshape CAF physical traits and dedifferentiate their phenotype at the cell-ECM axis. These platforms hold significant promise for engineering CAFs, aiming to restrain tumor progression *ex vivo*.

Building upon the earlier discussion highlighting CAF morphological features as crucial markers for their subtypes, the profound impact of ECM nano-topography on cell morphology presents an enticing opportunity. Utilizing nanopatterned substrates for precise control over CAF morphology and potential reprogramming to their original lineages emerges as an exciting strategy. Studies employing micropatterned substrates have demonstrated effective reprogramming of fibroblasts toward induced pluripotent stem cells and epithelial lineages.^190–193^

While nanopatterning precisely controls cell shape, its indirect influence on cytoskeletal arrangements governing contractility is a challenge. A recent study led by Cabezas *et al.* highlights that nanoscale anisotropic features within a patterned matrix exert direct control over actin fibers within confined shapes, offering a programmable approach within defined geometries.^190^ Moreover, mechanical reprogramming of fibroblasts through laterally confined micropatterned substrates induces a shift toward stem-cell-like phenotypes, reducing DNA damage and enhancing cytoskeletal gene expression and actomyosin contractility.^194^ Overall, utilizing nanopatterned substrates combined with biomaterials that mimic the cellular niche offers a powerful platform for the precise manipulation of the geometrical characteristics of CAFs. This advanced approach holds promise for directing the reversion of CAFs to their original phenotypes.

Incorporating the CAF study within the context of biomaterial environments paves the way for therapeutic innovations. By exploring the capacity of biomaterials to influence CAF phenotypes and functions, we can pinpoint novel therapeutic targets and craft innovative strategies. By Bridging foundational research with clinical possibilities, this integration transforms insights gained from *in vitro* models into actionable therapeutic interventions. The potential to develop treatments that specifically alter CAF activity, thereby hindering tumor growth and enhancing patient prognosis, is an exciting frontier in cancer therapy.

### D. Bridging the gap between biophysical and molecular markers

CAFs exhibit remarkable heterogeneity, yet they share several molecular markers that confer distinct biophysical characteristics, such as morphology and motility, influencing tumor behavior. Key markers include α-SMA, FSP-1/S100-A4, FAP, tenascin-C, PDGFR-α/β, CD90, MMPs, MHC-II, and podoplanin (PDPN), which are indicative of the CAFs’ role within the TME.^8,10^

#### 1. α-SMA: A model for understanding CAF phenotypes

α-SMA is a prominent CAF marker, closely associated with enhanced fibroblast contractility and ECM remodeling. This reflects CAFs’ contractile phenotype, as previously discussed regarding their morphological variability and motility characteristics.

The spatial distribution of α-SMA significantly affects fibroblast functions and contractility. A recent study by Kwartler *et al.* emphasizes that the nuclear accumulation of *α*-SMA in smooth muscle cells markedly enhances the expression of contractile genes.^195^ Importantly, *α*-SMA, when polymerized into stress fibers, not only induces contractility but also offers valuable insights into the evaluation of its localized position and correlation with morphological features. These biophysical traits are particularly significant in *α*-SMA-positive CAFs (*α*-SMA + CAFs), also known as myCAFs, which are crucial in ECM remodeling and force generation.

Furthermore, a study led by Barbazan *et al*. reveals that *α*-SMA-expressing CAFs, characterized by high contractility, actively compress cancer cells by forming an actomyosin ring.^192^ This compression is achieved through a coordinated contraction within the *α*-SMA expressing CAF layer. This study highlights the multifaceted role of α-SMA in orchestrating both structural and functional changes in CAFs.

#### 2. FSP-1 and mechanotransduction

We have reexamined the significance of the DDR2 gene in CAF mechanotransduction. In a study using FSP-1^cre^ mice, deletion of the DDR2 gene in FSP-1-expressing CAFs led to altered extracellular matrix (ECM) architecture and reduced tumor stiffness. It impeded lung metastasis, indicating that FSP-1 positive CAFs influence ECM mechanics and metastatic outcomes through the DDR2 pathway.^57^ Southern *et al.* further expounded on the role of FSP-1(S100A4) in myofibroblast trans-differentiation and fibrosis, where FSP-1 facilitates the reorganization of actomyosin, contributing to a contractile phenotype.^196^ The subtleties of FSP-1 positive CAFs necessitate further detailed investigation to elucidate their roles in mechanotransduction. This process highlights the importance of FSP-1 in the mechanical and functional evolution of CAFs. Nonetheless, it emphasizes the need for comprehensive research into FSP-1 positive CAFs, which could reveal further insights into their mechano-effector activities or their developmental lineage within the CAF population.

#### 3. FAP and ECM remodeling

Lee *et al.* highlighted the role of FAP-positive CAFs in ECM remodeling, which orchestrates the alignment of collagen fibers to foster an environment conducive to cancer invasion.^197^ The study elucidates the mechanotransduction function of FAP in cancer progression, highlighting how the FAP-positive CAF-derived matrix can enhance cancer cell invasion engaging β1-integrin/FAK.

### E. Challenges and future directions in developing biophysical-based therapies for CAFs

Developing biophysical properties-based therapies for CAFs poses significant challenges. One main obstacle is the variability within CAFs; they show different behaviors and have varied roles in the TME. Precisely targeting CAF subpopulations with unique biophysical traits may unlock effective therapeutic interventions. This would involve studying the mechanical, flow-related, and shape-related properties of CAFs using tools like magnetic tweezers, rheometers, and AFM-based methods (Fig. 3).

To better understand the roles and types of CAFs, we should consider both biochemical and biophysical information (Fig. 3). Future directions in this field involve identifying novel biophysical targets and devising innovative strategies to modulate CAF behavior. This could include investigating new techniques like drug delivery inspired by cell mechanics and incorporating biophysical cues into combination therapies. However, successfully implementing these strategies requires an integrative approach, employing a diverse set of mechanobiological tools to unravel the inherent mechanical behavior of CAFs.

## V. CONCLUSION AND IMPLICATIONS FOR FUTURE RESEARCH

While the potential of biomolecular markers has been extensively explored, the biophysical attributes of CAFs, such as those impacted by IFF and the associated mechanotransduction within the TME, bring a compelling dimension to our understanding of cancer progression. Integrating both biomolecular and biophysical characteristics affords a more holistic perspective of CAF subtypes and their multifaceted roles within the TME. This integrative approach goes beyond mere geometric measurements, encompassing CAF morphology, motility, mechanical properties, and biomolecular markers. Such an enhanced analytical framework promises to improve the precision in identifying and targeting specific CAF subpopulations therapeutically (Fig. 3).

Exploring the biophysical interactions of CAFs within the TME, particularly their role in CAF-ECM remodeling and intercellular communication, offers novel opportunities for therapeutic strategies. Incorporating insights on the role of fluid dynamics, such as IFF, is instrumental in dictating CAF phenotype and function, revealing a profound influence on ECM remodeling, CAF activation, and subsequent tumor cell interaction and invasion. Therapies targeting CAF-ECM interactions or mechanosignaling pathways have shown promise in reducing CAF activation and restraining tumor progression. In addition, the insights from the physical interactions between CAFs and inflammatory cells could provide novel modalities to modulate immune responses within the TME. These approaches, however, must be carefully optimized to avoid disrupting the intricate balance within the TME.

Innovative approaches, like biomaterial-based platforms, offer precise control over the phenotypic behavior of CAFs by altering the mechanical properties of the ECM and TME. These platforms can potentially shift the balance from pro-tumorigenic to anti-tumor activities. Still, they necessitate a deep understanding of the complex interactions within the TME to ensure efficacy and safety in clinical applications. Yet, these strategies must be developed with an awareness of the delicate balance within the TME and the possibility of unforeseen consequences.

The complexity of the TME and the consequential role of CAFs underline the necessity for an interdisciplinary approach. A collaborative effort that unites diverse fields ranging from oncology and mechanobiology to materials science and bioengineering is essential for constructing a comprehensive understanding of the TME. Such a multidisciplinary strategy is not merely advantageous but essential for unraveling the subtleties of CAF subtypes and exploring innovative therapeutic paths.

Ultimately, a balanced perspective that recognizes both the potential benefits and the inherent risks is critical for advancing cancer treatment. Interdisciplinary collaboration will be key in developing safer, more effective cancer therapies that enhance patient outcomes. By embracing this approach and integrating both biomolecular and biophysical insights, we can progress toward demystifying the nuances of CAF functions and unlocking new avenues for combating cancer.

## Data Availability

Data sharing is not applicable to this article as no new data were created or analyzed in this study.
